# Observations upon the Physiology of Audition

**Published:** 1858-02

**Authors:** A. M. Parker

**Affiliations:** Campbell Co., Ga.


### ARTICLE II.

*Observations upon the Physiology of Audition.* By A. M. PARKER, M. D. of Campbell Co., Ga.

In tracing the distinguishing characteristics of animals and plants, the physiologist is forced to admit that conciousness belongs alone to the former, and again all animals however, infe.

rior, are necessarily endowed with special sensation. All animals then may take cognizance of their own existence, and to some extent the external world, and may use the means they possess to perpetuate their existence and reproduce their species. Although the organs of special sensation in a number of classes have not as yet been detected, still it is rational to conclude that they do exist.

The oyster makes manifestations of its sensibility to light; yet no special organ of vision has been detected in that ani mal. Who but a naturalist or physiologist would ever have thought of looking in a grass-hoppers legs for its eyes? May not the vibrating cilia of a Protozoon possess in addition to their locomotive property, the property also of receiving and conveying sonorous vibrations to the sensorium, or have the sense of feeling, seeing, smell or taste? It is just and philosophical to conclude that all animals are endowed with the faculty of hearing; however the function may be performed, it is nevertheless the fulfillment of the design of the great Physiological Lawgiver, who never wrought in vain. As we ascend in the scale of animal existence the organ becomes more and more complex, till in man it arrives at its perfection. And thir complexity of contrivance in the ear of man is doubtless intended by the great Author of nature to give him a greater power of discriminating sounds than the rest of the animal creation.

It is to be regretted that all the researches of science in Physiology and acoustics have failed to furnish to the scientific world, a satisfactory theory in relation to the uses of the various parts, of the organ of hearing. It is not so with the eye. The manner in which the rays of light are let fall upon the retina so as to imprint the visual object is fully and satisfactorily exemplified in an artificial eye. And it was not without the laws of the refraction of light in different media that the laws of vision were explained. Here all the conditions could be imitated by art. And a like imitation, of the conditions in the phenomena of sound will be requisite to explain all the uses of an organ of hearing. Neurological, nor Pathological nor comparative anatomical research, isolated or combined, will ever be able to solve the whole of the question without a further developement in acoustics. Sonorous vibrations have the most various directions and the most equal rate of succession, are transmitted by all media without modification, however numerous their lines of intersection; and wherever these fall upon the auditory nerve they must cause the sensation of corresponding sounds. Yet withal we have a theory which must pass for the true one till the true one is discovered. It is probable that the various parts are adapted each to receive impressions (speaking of the internal ear) of a particular character; for instance, the directions would be indicated by one part of the organ, the pitch of sounds by another.

The essential part of an organ of hearing is a nerve endowed with the peculiar property of receiving and conveying sonorous vibrations to the seat of consciousness. In some of the invertebrate animals, the nerve is distributed to a membrane lining a little cavity in the solid framework of the head without any external opening. The sound then can only be propagated through the solid walls. But in the higher forms of this apparatus there is a small aperture covered with a membrane upon which the external medium can at once act. Thus we see, in the cavity, the vestibule of higher animals, and in the membrane the fenestra ovalis. In the lowest byclostome fishes a single anular passage proceeds from the vestibule which may be considered a semi-circular canal and the auditory nerve distributed to its lining membrane. In the Lamprey we find such canals. Ascending higher, we find the three semi-circular canals. Otolithes are found in fishes and some rudiments of a cochlea. It is said that in several tribes of fishes, the organ of hearing has a peculiar relation with the air bladder, which appears to be intended for a similar purpose to the Eustachian tube of higher animals. In the true reptiles a tympanic cavity is added, and the chain of bones and a rudiment of a cochlea. In Amphibia, frequently no tympanic cavity is found. But when there is a distinct tympanic cavity, there is also a Eustachian tube connecting it with the fauces. The tympanic cavity in true reptiles possesses the fenestra ovalis and fenestra rotundum. In birds, the cochlea instead of being spiral, is nearly straight, and the tympanum communicates with the cavities of the cranial bones, and thus adds to the intensity of sounds by resonance. In all the mammalia, the internal ear is generally found upon the same plan as it presents in man. The Labyrinth in some of the higher Vertebrata, contains no Otolithes. In man we have then, the external ear, the meatus externus, the membrana tympani, the tympanic cavity, the chain of bones, the fenestra ovalis and fenestra rotundum, the vestibule, the three semi-circular canals and cochlea, which are the bony and membranous labyrinth upon which is spread the auditory nerve continuing to or from the brain which is the great seat of the mind and consciousness.

It is necessary to consider briefly some of the means by which sounds are propagated :

1st. By Reciprocation, as when two strings of equal size, length and tension are placed along side of each other, and one of them be sounded with a violin bow, the other will be thrown into reciprocal vibration.

2d. By Resonance, as when a tuning fork in vibration is placed upon a sound board, the latter divides itself into parts, each of which reciprocates and increases the sound of the tuning fork, though the sound board have no definite fundamental note, the same sound board acting equally well for a tuning fork of several degrees of pitch. But when a smaller body is used for resonance, it is essential there should be a relation between its fundamental note and that of the sounding body; otherwise, no distinct resonance is produced. Thus, if a tuning fork in vibration be held over a column of air in a tube of such length that the same note would be given by its vibration, its sound will be reciprocated. But if the sound of the air in the tube would by its fundamental note produce an octave below this, the sound will, by separating into segments, be that of resonance. Or the column of air may divide itself into parts so as to sound the 12th, 15th, 17th, 19th, 20th or 22d, each or all of these sounding at the same time in harmony with the fundamental note itself, and producing what is called "grave harmonies." It is upon the knowledge of these laws of acoustics that much of the beauty of harmony depends. And it exhibits too, that as the divisions of the musical scale are founded in nature, and not in the mere arbitrary rules of art, so is the organ of hearing in man wisely adapted to reciprocate and be delighted with the sounds of harmony or melody. Paganini, the violinist, was thus enabled to play regular harmonies upon a single string of the violin. Man is not the only animal that appreciates music. We have heard of a horse that seemed to take delight in hearing his master's violin.

3rd. Vibrations of Conduction are those by which sounds are ordinarily propagated; and these may be more rapidly through solids, next through liquids, next through æriform, and not at all through a vacuum, (Muller's experiments in Carpenter's Elements of Physiology.) The intensity of sound is greatly diminished in passing from a gaseous to a liquid medium; but if a tense membrane be between the one and the other, the sonorous undulations are communicated from the former to the latter with great intensity. This explains the process of hearing in those animals which receive the sounds in or through air, but have no tympanic apparatus. Sonorous vibrations are communicated without any perceptible loss of intensity from air to water when to the membrane forming the medium of communication there is attached a short solid body which occupies the greater part of its surface, and is alone in contact with the water; applicable to amphibia and animals living in air, but destitute of tympanic apparatus.

A small solid body fixed in an opening by means of a border of membrane, so as to be moveable, communicates sonorous vibrations from air on one side to water, or the fluid of the labyrinth, on the other, much better than solid media not so constructed.

The membrana tympani is rendered tense or lax by the re flex action of the tympani muscles; on the same principle it is supposed that the iris of the eye contracts or dilates according as the light is intense or not, and by this means the membrane can be made to reciprocate grave or acute sounds. It receives the sonorous vibrations and communicates them to the chain of bones and by these they are conducted more directly to the fenestra ovalis, but there is also at the same time a powerful resonance in the cavity of the tympanum which has an additional force by virtue of the mastoid cells. In this, not only the fenestra rotundum and fenestra ovalis are affected by the vibrations, but also the external walls of the labyrinth. No wonder that the fluid of the labyrinth receives the sonorous impulses, however slight, when it is considered what powerful means are at work in the communication of sounds.

It is supposed that the semicircular canals have for their peculiar function the reception of impressions by which we distinguish the direction of sounds, and that the cochlea enables us to estimate the pitch. But as these are merely hypothetical opinions, of which there is no satisfactory proof, it is deemed unnecessary to say more ; nor is it probable that the more special functions of these will ever be discovered, except, perhaps by some experimenter, who must be familiar with the laws of sound, and must imitate the ear in all its physical parts.—Locked up as these are in the petrous part of the temporal bone, they are out of the reach of satisfactory experiment. The object of the Eustachian tube appears to be the maintenance of the equilibrium between the external and internal air, by which the vibrations are rendered free. It also serves the purpose of conveying away the mucus secretions from the tympanum by the vibrating cilia lining its mucus surface, to the cavity of the throat. The cartilage of the external ear which is continuous with that of the meatus, by the inequalities of its surface, reflects and conducts the sound ; but this is more properly the function of the concha ; by all these means, however, it is condensed and conducted to the membrana tympani. The functions of the meatus are three-fold: 1st to convey the sonorous vibrations directly to the membrane ; 2d, the vibrations are conveyed along the walls by conduction ; 3d, the air which it contains like all insulated bodies of air, increases the intensity of sound by resonance.

It is upon the application of the principles of sound or a knowledge of the laws of sound rather, that the stethescope is made. This instrument is intended to insulate or circumscribe a particular part of the body in auscultation, rather than to increase the intensity of sound, which it does but slightly if at all. It is a matter of wonder that even with our imperfect knowledge of acoustics, the instrument has not received a greater degree of attention, and, consequently of improvement· Why, with our knowledge of resonance may not an instru ment be constructed by which the intensity of sound may be greatly augmented? Any such instrument that would materially assist the physician in forming his diagnosis of the viscera would be a rich boon, and would crown the inventor with immortal honor.

Sound is propagated through the air at the rate of 1142 ft. per second, while light is transmitted at the rate of 200,000 miles per second. While light therefore travels from the sun to the earth 95,000,000 of miles in eight minutes of time, sound would require (all contingencies removed) several years, say 13½ years in traveling the same distance. In this comparison we perceive at once that vision is affected ordinarily long before sound could reach the ear. With these phenomena every one is familiar in the ordinary occurrencies of life.

A slight impulse communicated to the auditory nerve, excites a momentory sensation of sound; but a series of such impulses is necessary to produce a musical tone, the acuteness or depth of which depends upon the degree of rapidity with which they are produced. A spring submitted to the action of a revolving toothed wheel will give a double impulse for each tooth that strikes it, and the graveness or acuteness is governed by the number of impulses, and not by the loudness or softness. The number of vibrations or impulses of a musical string is according to its length, M. Savart found by experiment that the range of acuteness or gravity of sounds or tones apprecia ble by the human ear was in the acutest, 24,000 vibrations pe second, and in the gravest tone 7 or 8 impulses per second.—In regard to the length of time which a sonorous impression remains upon the ear it is uncertain. M. Savart found that several teeth might be removed from the toothed wheel with out any perceptible brake in the sound.

It would depend upon the natural or acquired acuteness or slowness in different individuals of their perception of sensory impressions, just as the rule varies on the same account in regard to vision. The power of distinguishing the direction of sounds and their distance also, is acquired by the attention which the mind involuntarily gives to certain variations in space, with which the young become more and more acquainted in proportion as the powers of voluntary motion and of mind becomes developed; and also, by the comparative loudness or softness of sounds with which he is familiar. The sensation or consciousness of sound occurs in the brain.

The subjective phenomena of sound have their origin somewhere in the substance of the brain or between it and the periphery, which are referred by the mind to the periphery, and the causes are frequently if not always obscure. On the first inhalation of sulphuric ether for its exhilarant effects, the writer of this experienced the delightful sounds of many tiny musical bells, which gradually died away as he imagined he sailed up without effort in the moon-lit air, whence descending back amongst his friends he alternated between high stepping and friendly manifestations till its effects wore off.

The distance at which sound may be heard depends upon the smallest amount of impediments, all of which being removed it can be heard over 100 miles. One instance of this is given in the history of the fatal siege of the Alamo, Texas.

The nearness of sound may be exemplified by irritating the short stiff hairs of the meatus. And painful loudness is felt when an insect encroaches in that sacred cavity. But encroachments of that character are much less frequent than they would be but for the unctious cerumen secreted by the follicles of the meatus and which is repugnant to the smell of insects. another instance of the nearness of sound, I conjecture, is the bursting of a bubble of cerumen and giving rise to "death-bell" as it is superstitiously called. Loudness of sounds may be borne with a much less degree of discomfort if there be no discord. Even in sounds moderately loud, discordant notes, by producing a jarring in the membrane, inflict pain and disgust, especially in a refined musical ear. Imagine twelve or fifteen locomotive engines in full blast, each whistle blowing its own note which discords with all the rest and you may have an idea of the painfulness of discord. It is more intolerable than the roaring cannon which may be heard ten times as far.—Another disagreeable sensation of sound is produced by scraping a dry reed with a knife, producing chilliness or "crawling of the flesh."

It would be interesting, and perhaps instructive to consider the usefulness of sound in its application to natural language, in man as well as the inferior animals. How the sounds with which man was familiar in his primeval state were gradually developed into natural or spoken language: how, by the use of hieroglyphics and signs, written language was developed. How "Young America's" were taunted by the "Old Fogies" of those olden times; how, despite those taunts, the improvements and developments in science, hitherto traditionary, became their own recorders in a written language; how then it became necessary for convenience sake that the essays of the learned were put in print; how, now language is made to get into the electric current to be silently conveyed to the opposite end of the telegraphic wire, where educated eyes are the ready interpreters; how, by these artificial sounds nation can converse with nation.

The majesty of sound was heard in chaos in the Creator's voice, when He said, "Let there be light, and there was light." Thus was *our subject* first honored, and thus was sound the honored agency of the Deity in the creation of that subtle agent about the nature of which philosophers quarrel. Ye philosophers cease your controversy about light, and tell me what is nervous influence? What is life? What is the soul of man? What is the resurrection? Ah! your reason may toil in vain! Look and bow to the Revelation of God. He hath power to take away your nervous influence with your natural life! But there is another influence, another life behind this, and mysteriously identified with it. It is that of the soul immortal. He that gave these and with them dominion over all the earth, hath power to reunite them in the Resurrection, element to element, function to function, if need be, atom to atom.

The superiority of man over the rest of the animal creation derives proof from a thousand sources. Whether we look at his superior anatomical structure, or the more complex and various powers of mind, or at the inmost recesses and capacities of the soul, all go to prove the truth of Revelation, where it is said that man is made in the image of God. Yet with all man's superior endowments, his wisdom is foolishness with God. And whether I behold man in his lordly domain, tilling the soil, navigating the ocean, or wielding the implements of art, scanning the depths of nature's laboratory, in the animal, vegetable and mineral kingdoms; or drawing out the long hidden mysteries of the heavenly bodies, where

> "Throughout the galaxy's extended line,
> Unnumbered orbs in gay confusion shine,
> Where every star that gilds the gloom of night
> With the faint tremblings of a distant light,
> Perhaps illumes some system of its own
> With the strong influence of a radiant sun."

In it all I but see a grand confirmation of the supremacy and glory of God.

